# *Salix* transect of Europe: records of willow-associated weevils (Coleoptera: Curculionoidea) from Greece to Arctic Norway, with insights from DNA barcoding

**DOI:** 10.3897/BDJ.8.e52881

**Published:** 2020-06-03

**Authors:** Roy Canty, Enrico Ruzzier, Quentin C Cronk, Diana M Percy

**Affiliations:** 1 Natural History Museum, Cromwell Road, SW7 5BD, London, United Kingdom Natural History Museum, Cromwell Road, SW7 5BD London United Kingdom; 2 Universtità degli Studi di Padova, Legnaro (Padova), Italy Universtità degli Studi di Padova Legnaro (Padova) Italy; 3 World Biodiversity Association Onlus, Verona, Italy World Biodiversity Association Onlus Verona Italy; 4 University of British Columbia, Vancouver, Canada University of British Columbia Vancouver Canada; 5 Natural History Museum, London, United Kingdom Natural History Museum London United Kingdom

**Keywords:** Salicophagy, salicivorous insects, Salicaceae, Curculionoidea, DNA barcoding, Europe, megatransect

## Abstract

**Background:**

Curculionid beetles associated with willow (*Salix* spp.) were surveyed at 42 sites across Europe, from Greece (lat. 38.8 °N) to arctic Norway (lat. 69.7 °N). DNA sequence data provide additional verification of identifications and geographic clustering.

**New information:**

In all, 73 curculionid species were collected from willows, of which seven were particularly abundant. The most widespread species were: *Acalyptus carpini* Fabricius, 1793 at 15 sites; *Tachyerges stigma* Germar, 1821 at 13 sites; *Phyllobius oblongus* (Linnaeus, 1758) at 11 sites; *Phyllobius maculicornis* Germar, 1824 at 10 sites; and *Archarius salicivorus* (Paykull, 1792), *Melanapion minimum* (Herbst, 1797), and *Phyllobius cf. pyri* (Linnaeus, 1758) all at nine sites. The mean number of curculionid species collected on willow at each site was 5.5 (range 0-14). Compared to chrysomelids, curculionids were richer in species but the species had relatively low average abundance. Widespread curculionid species appear to have scattered and patchy observed distributions with limited geographical structuring in our data. However, deeper sampling (e.g. over multiple seasons and years), would give a better indication of distribution, and may increase apparent geographical structuring. There is some site-to-site variation in colour in a few taxa, but little notable size variation. DNA barcoding, performed on some of the more common species, provides clear species clusters and definitive separation of the taxonomically more challenging species, as well as some interesting geographic insights. Our northernmost sample of *Phyllobius oblongus* is unique in clustering with Canadian samples of this species. On the other hand, our samples of *Acalyptus carpini* cluster with European samples and are distinct from a separate Canadian cluster of this species. We provide the first available DNA sequences for *Phyllobius thalassinus* Gyllenhal, 1834 (Hungary).

## Introduction

Weevils (Coleoptera, superfamily Curculionoidea Latreille, 1802) are a hyperdiverse group of phytophagous and mycophagous insects. They are divided into several families of which the principal is the “true weevil” family Curculionidae Latreille, 1802. This in turn is divided into numerous subfamilies (Oberprieler et al. 2007, Gillett et al. 2014). Weevils have evolved to take advantage of a wide variety of plants and plant organs. The plant host range of the group spans most seed plant groups and many ferns. In their use of plant niches they have evolved both endophagous (internal feeding) and ectophagous (external feeding) lineages. Species utilise stems (including trunk borers of economic importance), leaves (including larval leaf miners) and reproductive structures (flowers, cones and seeds) (Marvaldi et al. 2002).

Weevils are generally narrowly to broadly oligophagous, with some extremely polyphagous species (Anderson 1993). Typically, species feed on either a limited range of unrelated plant species, or on a closely related group of species. A few species are monophagous. A large number of species have been recorded feeding on *Salix* spp. (willows: Salicaceae) (e.g. DBIF 2008, Hoffman 1958). These may be divided into four types based on host preference:

1. genus specialists (*Salix* only), such as many species of *Isochnus* Thomson, C.G., 1859, *Tachyerges* Schönherr, 1825 and *Dorytomus* Germar, 1817.
2. clade specialists, i.e. restricted to *Salix* L. and its sister genus *Populus* L. (poplars and aspens), such as *Dorytomus taeniatus* (Fabricius, 1781);
3. transgressive specialists, which feed on *Salix* and a very limited range of unrelated species, such as *Acalyptus carpini* (Fabricius, 1793) which feeds on *Salix* and *Carpinus* L. (Betulaceae); and
4. generalists, such as *Polydrusus pterygomalis* (Boheman, 1840) which has host records in the plant families Fagaceae Dumort, Ulmaceae Mirb., Salicaceae Mirb., Pinaceae Lindley, Rosaceae Juss. and Betulaceae Gray.

Willow feeding weevils utilise many parts of the host. Some, such as *Tachyerges*, *Isochnus* (Anderson 1989), and *Rhamphus* Clairville, 1798 have leaf-mining larvae. Some are inquilines in sawfly (Hymenoptera: Tenthredinidae) galls or leaf rolls of the genera *Euura* Newman, 1837, *Phyllocolpa* Benson, 1960 and *Pontania* Costa, 1859 on willow. The beetle larvae feed on the gall tissue and frequently destroy the gall-maker (Caltagirone 1964, Kopelke 2003). An example of a gall inquiline in *Pontania* galls is *Melanapion minimum* (Herbst, 1797) (Brentidae) (Askew and Kopelke 1988). Weevils also bore into stem tissue, and a Salicaceae specialist stem borer is *Cryptorhynchus lapathi*, which is described as a serious pest of commercial basket willow plantations in the UK (Smith and Stott 1964). This species has also been introduced into British Columbia (Canada) where it is affecting native willows and hybrid poplar (*Populus*) plantations (Broberg et al. 2002, Harris and Coppel 1967, Johnson and Johnson 2003).

There are many challenges in establishing the extent of host preference in phytophagous insects, including teasing apart complex environmental cues, and in some cases experimental results are not apparent in the field. In laboratory experiments, *Orchestes fagi* (Linnaeus, 1758) (a leaf mining weevil and *Fagus* L., Fagaceae, specialist) made feeding holes in a number of offered hosts, including *Salix*, but *Fagus* was overwhelmingly preferred (Bale and Luff 1978). The wood-boring weevil *Cryptorhynchus lapathi* (Linnaeus, 1758) shows olfactory preferences for some willows over others (Broberg et al. 2005) although in the field there is little evidence of differences in incidence of attack (Broberg et al. 2001). The presence or absence of phenolglycosides in different willow species (Hegnauer 1973) has also been shown to influence weevil host preference (Rowell-Rahier 1984). However, there are still many unanswered questions and many untested influences on weevil-host interactions.

As well as confirming taxonomic placement and highlighting population structure not apparent in morphology alone, a molecular component to taxonomy has increasingly become routine, with the use of DNA barcoding (Hebert et al. 2003a, Hebert et al. 2003b, Tautz et al. 2003). It is now well established that, in many animal groups, sequencing mitochondrial cytochrome oxidase subunit 1 (COI) and to a lesser extent, but increasingly common, cytochrome B (cytB), provides a straightforward way of gaining both taxonomic and geographic insight (Canty et al. 2019, Wonglersak et al. 2017).

As part of a broader study on lowland willow communities across Europe we investigated occurrence and abundance of weevils (Curculionoidea) associated with willows (*Salix* spp.) over a broad geographic scale. Weevils were collected from 42 willow stands covering the length of a north-south megatransect from Greece to Arctic Norway. This megatransect has been previously described in Cronk et al. (2015). This and previous studies from the same megatransect (see Biodiversity Data Journal series: *Salix* transect of Europe) provide occurrence data as a "snapshot" during a single sampling event and these data are intended to lay the ground work on which subsequent sampling across seasons, years, and taxa can build a more detailed overall picture to indicate historical changes through time.

## Sampling methods

### Sampling description

**Collecting methods**

Willow-associated beetles (in this context refers to all samples from *Salix* spp. at a particular site) were collected (by ER and DP) at every site, as described by Canty et al. (2016). Details of the sites and the method of their selection have been given in previous papers (Canty et al. 2016, Canty et al. 2019, Cronk et al. 2015). Briefly, rapid biodiversity sampling (42 localities) was employed over a megatransect from Greece to Arctic Norway. This route was driven in two stages in the spring of 2015. Stops were made approximately every 100 km to locate and sample a stand of willows (Table 1). Roughly one hour of sweeping was carried out per site, covering all the willow taxa present at a site. Beetle samples were field-collected directly into 90% alcohol. The willow species present and the willow voucher herbarium specimens are detailed elsewhere (Cronk et al. 2015). For the purposes of this study, all curculionids present at a site, whether collected from one or more willow species, are pooled. All material is deposited in the Natural History Museum, London (BMNH). Details of the environmental conditions (relative humidity and temperature) and time of day at collection have already been given for 41 of the sites (Canty et al. 2016). This paper includes an extra site (site 42); site 42 (Table 1), which was sampled at 16.00 hrs and the following environmental conditions were recorded: relative humidity (rH) = 54% and temperature (t°C) = 13.8.

**Specimen examination and analysis**

Procedures were similar to those used in Canty et al. (2016). For identification (by RC) the following works and resources were consulted: Morris (1997), Morris (2002), Morris (2012), Die Käfer Europas (Lompe 2016) and the species list from Volf et al. (2015). For each locality, specimens were sorted into broad morphospecies likely to correspond to biological species. These taxonomic units were then identified, and numbers of individuals of each taxonomic unit determined. Pending further critical taxonomic study, some misidentification is possible, and some identifications are tentative (indicated with cf.). However, the DNA analysis (below) did enable additional confirmation of species identification for some of the commoner species and related problematic specimens, as well as information about infraspecific genetic variation.

To assess morphological variation, eight of the more abundant species were chosen as “focal species” for further study. These were: *Acalyptus carpini, Isochnus foliorum, Isochnus sequensi, Melanapion minimum, Phyllobius maculicornis, Phyllobius oblongus, Rhamphus pulicarius, Tachyerges pseudostigma*. One to three individuals per site, from each four to six sites were selected for detailed examination. A Zeiss Stemi DV4 dissecting scope was used for morphological observations. Measurements were taken using a Minitool miniature measuring scale (range: 5mm; precision: 0.1mm). Colours were determined by visual matching under diffused daylight, using the standard RHS colour chart (Royal Horticultural Society 2007). The RHS numerical colour codes were converted to common language colour names using a standard mapping (UPOV 2013). Photography utilised a Canon EOS 700D camera mounted on a Leica MZ12.5 stereomicroscope. Images were taken via a computer with the Canon EOS 700D Utility Remote Live View programme. Multiple images were taken to enhance depth of field and combined using Helicon Focus (version 5.3) stacking software.

**Molecular methods and analysis**

Molecular data was obtained for two mitochondrial regions cytochrome oxidase subunit 1 (COI) and cytochrome B (cytB) for a subset of samples (1-6 samples) for each of the aforementioned focal curculionid species (*Acalyptus carpini, Isochnus foliorum, Isochnus sequensi, Melanapion minimum, Phyllobius oblongus, Phyllobius maculicornis, Rhamphus pulicarius, Tachyerges pseudostigma*) and some related specimens (*Phyllobius arborator, Phyllobius thalassinus, Isochnus flagellum, Tachyerges stigma*) (Table 2). DNA was obtained from material preserved in ethanol, and protocols for DNA extraction, polymerase chain reaction and sequencing follow those described in Percy et al. (2018). The COI sequences were aligned with published sequences from GenBank (Table 3) to provide confirmation of identification and estimate sequence divergence across transect sites. The reported genetic distances and the phylogenetic analysis with bootstrap support (1000 replicates) were obtained using neighbour-joining (NJ) analyses with uncorrected (p) distances in PAUP* (Swofford 2003). Sequences generated in this study are deposited in GenBank under accession numbers MN607603 - MN607645 (Table 2).

## Geographic coverage

### Description

**Geographical patterns and phylogeography of the common species**

**Figure 1.**
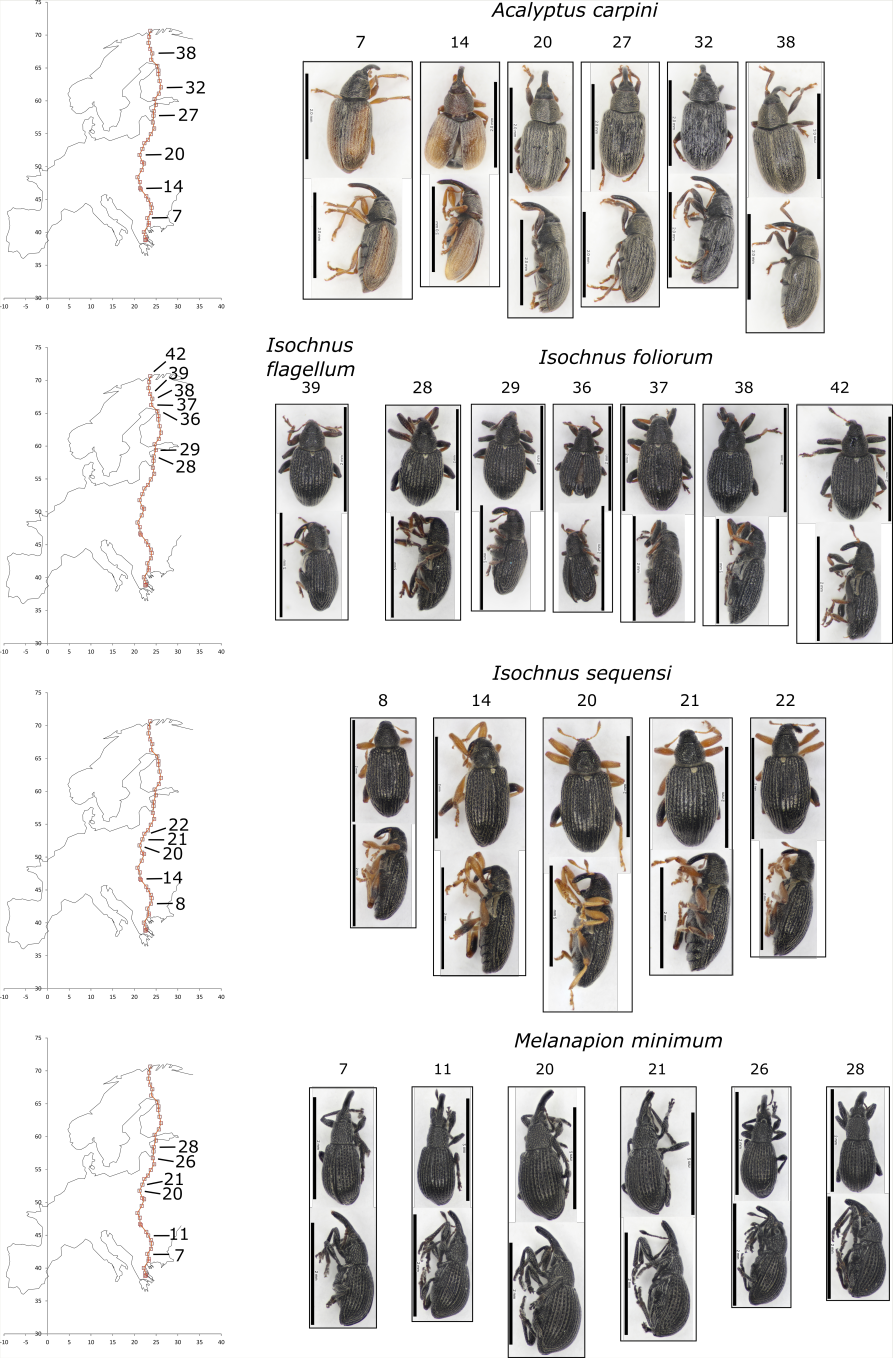
Images of representative examples of common species from different populations. Species: *Acalyptus carpini, Isochnus flagellum, Isochnus foliorum, Isochnus sequensi, Melanapium minimum*. Sample site localities are indicated on adjacent maps (left). Scale bars = 1 mm.

**Figure 2.**
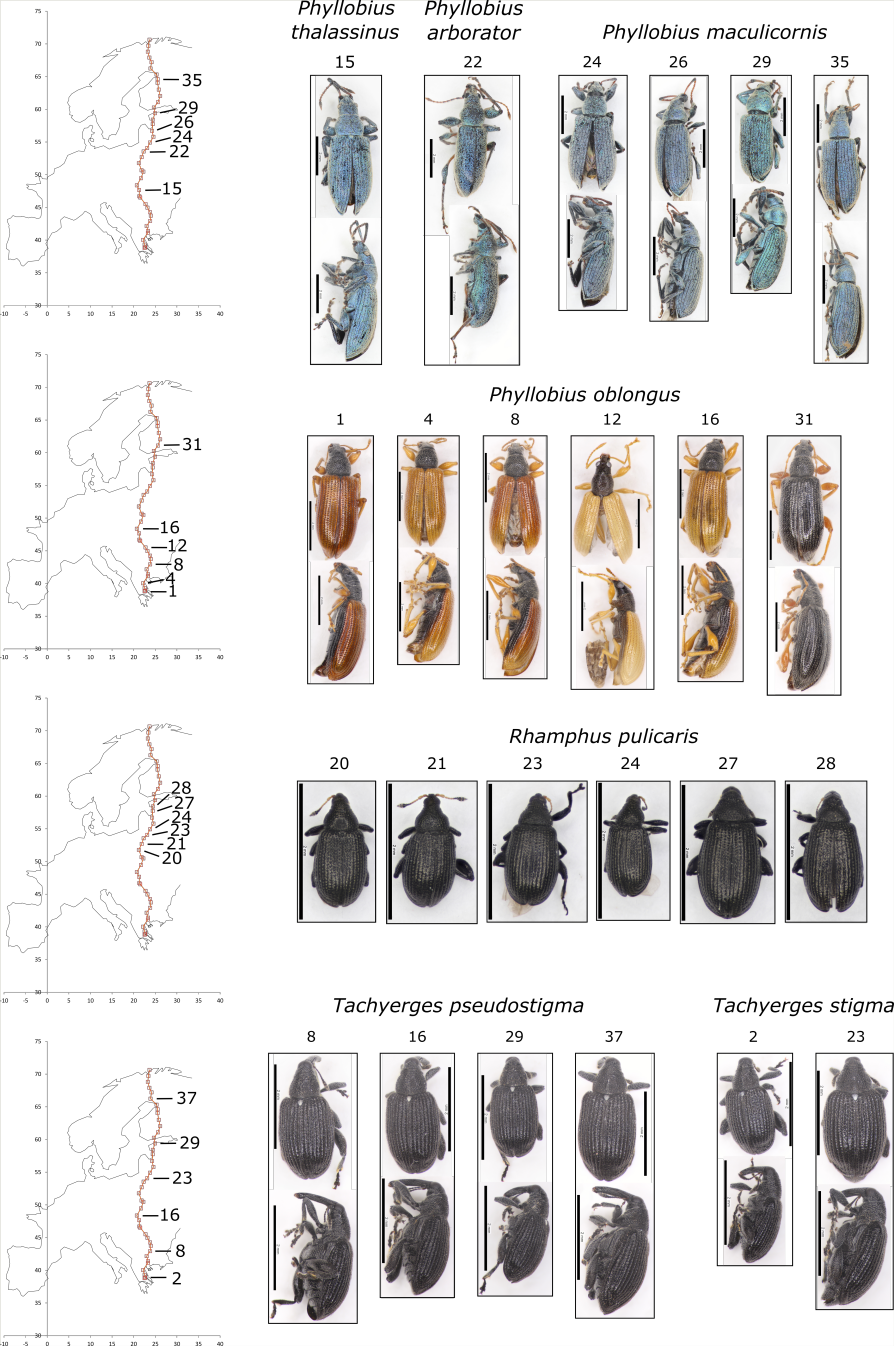
Images of representative examples of common species from different populations. Species: *Phyllobius thalassinus* (see molecular analysis), *Phyllobius arborator*, *Phyllobius maculicornis, Phyllobius oblongus, Tachyerges pseudostigma, Tachyerges stigma, Rhamphus pulicarius*. Sample site localities are indicated on adjacent maps.

**Figure 3.**
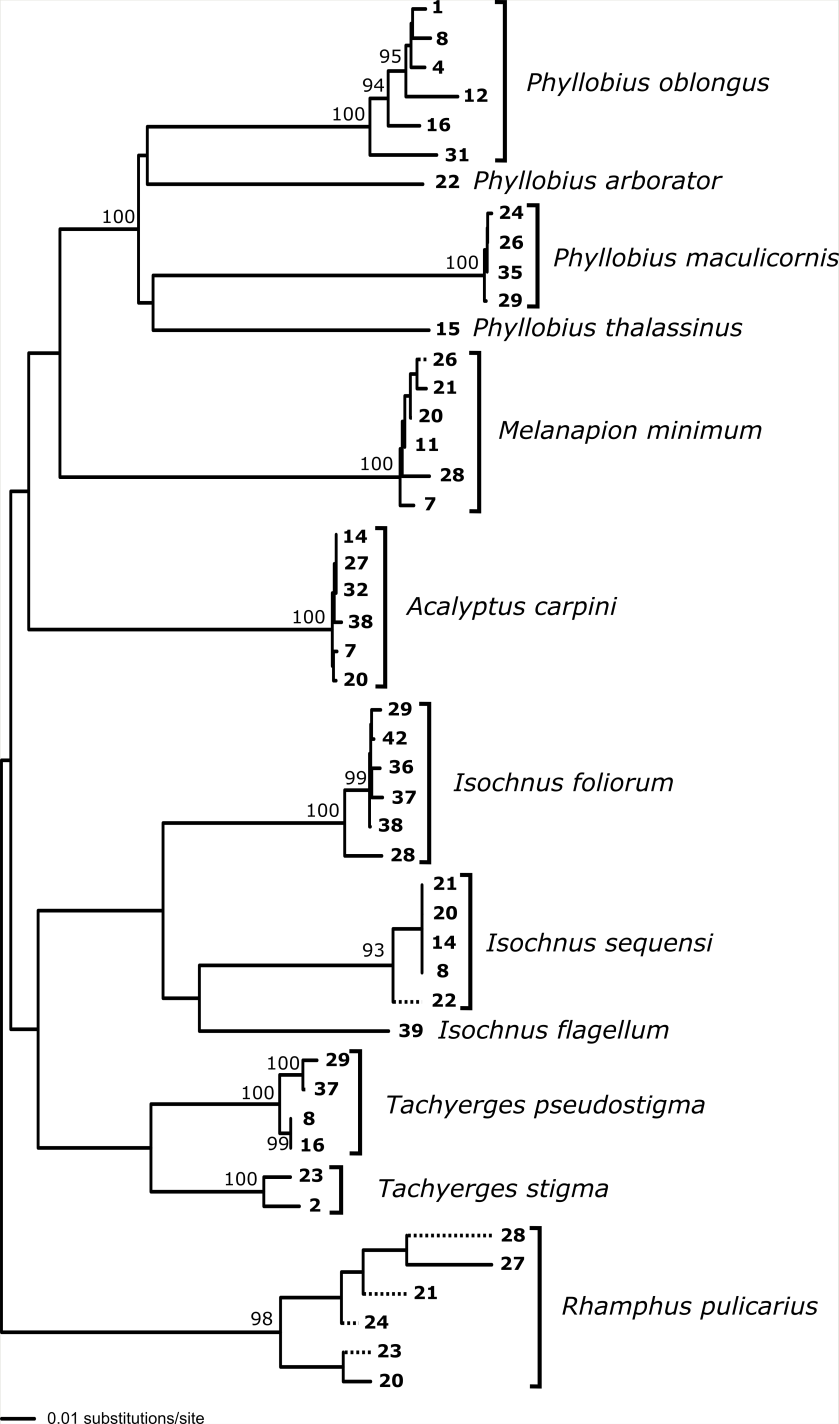
DNA analysis of Curculionoidea using COI and cytB sequences for transect samples only. Node support shown only for nodes with > 90% bootstrap support.

**Figure 4.**
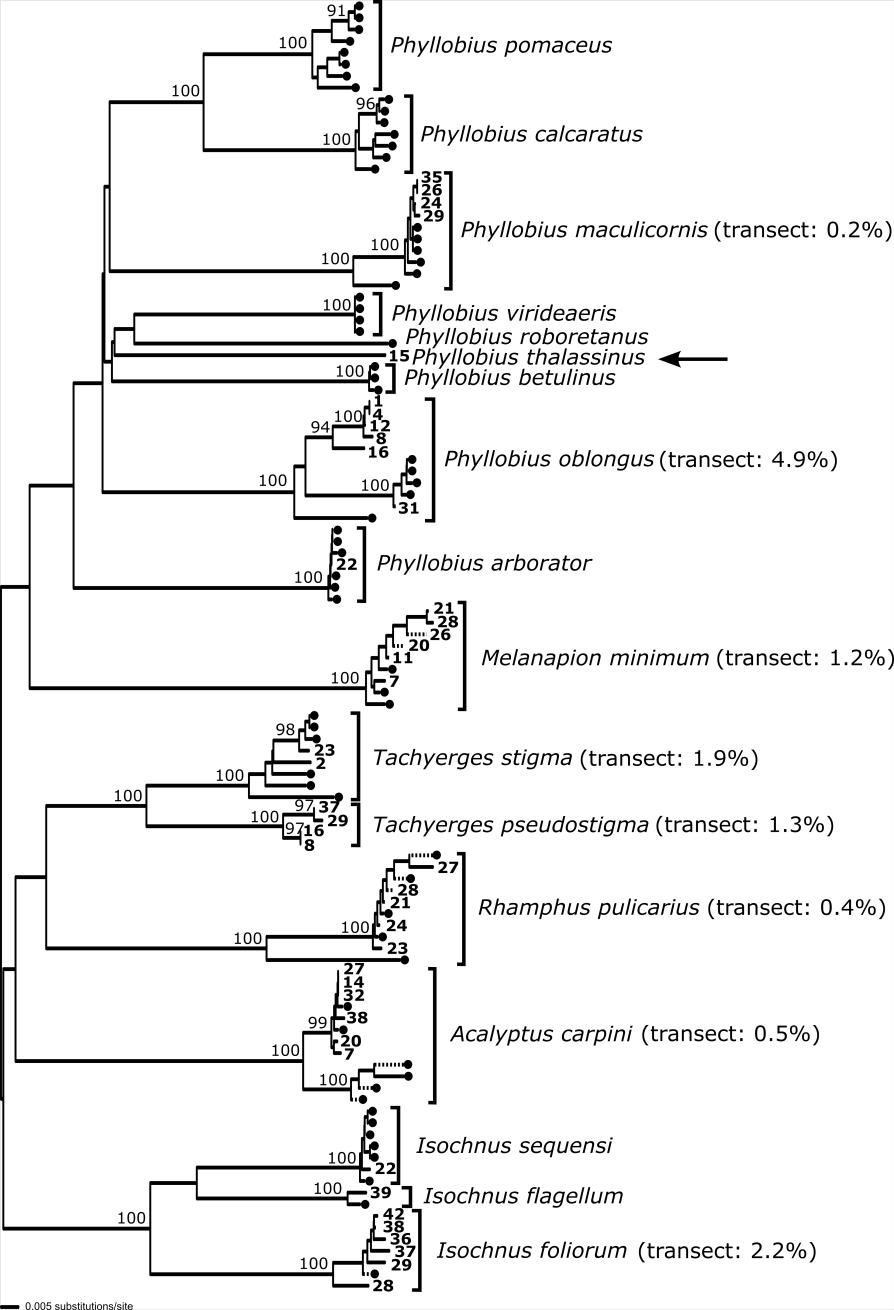
DNA barcoding analysis of Curculionoidea using COI sequences generated in this study and samples from GenBank. Sequences from this study show the site number, and those obtained from GenBank are indicated by a black circle (GenBank accessions given in Table 5). Arrow indicates *Phyllobius thalassinus* from site 15. Node support shown for nodes with > 90% bootstrap support. Maximum intraspecific divergences (%) are shown for transect samples estimated using uncorrected (p) distances (see methods).

Of those species that are present at a sufficient number of sites to allow assessment of geographical patterns, many are very widespread (Table 4, Figs 1, 2). Examples are *Acalyptus carpini* and *Tachyerges stigma* (our record being the most southerly published for this species), both occurring in a scattered fashion from Greece to Finland. However, it is evident that, in our sample at least, there are some species with a more northerly distributional bias and some more southerly. Most striking is the difference between two closely related willow-specialists: *Isochnus foliorum* (Müller, O.F., 1764) and *Isochnus sequensi* (Stierlin, 1894). The former we mainly found in Finland and Norway and it is most abundant in the northernmost site (42); the most southerly sample from Estonia (site 28) has a more divergent haplotype (Fig. 3). The latter has a non-overlapping, more southerly distribution in our samples, centred on Poland and occurring as far south as Bulgaria (site 8); and the most northerly sample has a more divergent haplotype. An *Isochnus* sample in Finland (site 39) DNA barcoded to *I. flagellum* Ericson, 1902, a species that did not appear elsewhere in our sampling (Fig. 4). A noteworthy feature is the presence of outliers in some species. For instance, while *Rhamphus pulicarius* is generally northern in our samples (Poland to Finland), we have an outlier in Greece (site 2). In contrast, while *Phyllobius oblongus* is southern in our samples (Greece to Hungary), we have an outlier in Finland, and this haplotype clusters apart from the southern individuals and together with samples from GenBank collected in Ontario (central Canada) (Fig. 4). In addition, two samples of *Phyllobius* Germar, 1824, not represented elsewhere in our sampling, barcoded to *P. arborator* (Herbst, 1797) (site 22); and we provide the first available DNA sequences for *P. thalassinus* Gyllenhal, 1834 (site 15) (Figs 3, 4).

### Coordinates

 and N 38.80007, E 22.4629 Latitude; and N 70.65234, E 23.66583 Longitude.

## Traits coverage

**Morphological variation**

Morphological variation within the common species is recorded in Table 6. We noted no particularly marked size variation within species. There was minimal intrasite colour variation within weevil species although some site-to-site variation, such as the lighter elytra colour in southern specimens of *Acalyptus carpini* (sites 7 & 14) versus the darker colour in central and northern specimens (sites 20-38; see Fig. 1). In addition, the northern specimen of *Phyllobius oblongus* (from site 31) already noted for the haplotype clustering with other boreal specimens from Canada) is notably darker than the southern European specimens (Fig. 2).

## Temporal coverage

### Notes

Collecting was conducted between April and June 2015 (see Table 1)

## Collection data

### Collection name

*Salix* transect of Europe: records of willow-associated weevils.**Species encountered and their relative abundance** - A total of 647 weevils were collected from 42 localities (including one locality, 20, that was collected at two times of year: 30 April and 11 June 2015). The two collecting events at site 20 are treated as two different “sites”: 20 and 20a. Three weevils (*Acalyptus carpini, Phyllobius oblongus* (Linnaeus, 1758), and *Tachyerges stigma* Germar, 1821) were most widespread, being found at 11 or more sites (Table 4). Next most widespread were *Archarius salicivorus* (Paykull, 1792), *Rhamphus pulicarius* (Herbst, 1795), and Phyllobius cf. pyri (Linnaeus, 1758), each at nine sites. The abundances per site of these six species are given in and together they make up a total of 214 individuals (around one third the total). A total of 74 species of weevil were recorded, although 36 of these were recorded at a single site (and 31 as a single individual only). It is possible that some of these latter are not willow feeders but are incidental by-catch. Generally, there is a strong correlation between number of localities and number of individuals (i.e. widespread species tend to be abundant when found). However, there are exceptions to this. *Polydrusus flavipes* (De Geer, 1775) was found at six sites (13, 20, 20a, 21, 28 and 31) but of the 82 individuals taken, 73 of these occurred at only one site (21). In contrast, *Archarius salicivorus* and *Archarius crux* were found at nine and eight sites respectively but only 14 individuals of each were taken. The average number of weevil species per site is 5.5 (range: 0-14) but it is clear that there is a lot of dispersion from that mean. Some sites proved to be “weevil hot-spots” with six sites having 12 or more species (11, 12, 20, 20a, 21, 28: in Romania, Poland and Estonia). On the other hand, four sites had only a single weevil recorded (3, 5, 34, 40: Greece, Finland and Norway) and in one no weevils were collected (9: Bulgaria). The differences in weevil richness may be due to intrinsic site factors (eg. quality of environment, land use, plant diversity) or to date of sampling and this is discussed below. In the case of the site with no weevils recorded (9), it is worth noting that this site (on the south bank of the R. Danube) was also lowest in willow diversity, having only *Salix alba* L. present (Cronk et al. 2015).**Occurrence and abundance** - In approximately 42 hours of sweep-net sampling (includes sweeping through foliage and knocking branches with net below) (c. 1 hour per site) we were able to recover 647 weevil individuals from *Salix* spp., belonging to 74 species. However, the fact that very many of these species were taken only as single individuals indicates that it is likely that we have only scratched the surface of total weevil diversity on willow and that further sampling at each site would have led to many more species being observed. However, although this is clearly far from a total inventory of willow-associated weevils in Europe, and it is possible that some species captured are not willow associated (i.e. by-catch), our study does show clearly which are the commonest willow weevils across the continent. Even the most common species in our survey have a scattered occurrence and they vary greatly in numbers of individuals per site. Thus it is likely that (with further sampling) the most widespread species could have been found at extra sites. The variation of abundance at different sites could be due to intrinsic site factors or to an interaction between sampling date, species phenology and local weather. This is underlined by the patterns at the only locality (20) that was sampled twice (in April as site 20, and June as site 20a), this locality is approximately mid-way along the transect. Combined samples (20 and 20a) had 17 species recorded, but only six species were present in both samples. The added information from DNA barcoding contributes to a more detailed picture of diversity and potential cryptic patterns such as the boreal *Phyllobius oblongus* sample. The sort of geographically extensive but time-limited survey reported here therefore represents a “snapshot” of beetle diversity across a wide area and is complementary to complete inventories of local areas conducted through the year. Its signal value is that it gives a vivid picture of the spatial heterogeneity of beetle occurrence.**Comparison with the Chrysomelidae** - It is instructive to compare our results for the curculionids with results from the same transect for chrysomelids. Curculionids and chrysomelids were co-collected so there can be no bias from sampling method or date. The chrysomelids tended to be more widespread and more abundant. The most widespread chrysomelid (*Crepidodera aurata*) was present in 27 localities, whereas the most widespread curculionid (*Acalyptus carpini*) was present in only 15 localities. Similarly, the most abundant chrysomelids (*Crepidodera aurata* and *Galerucella lineola* (Fabricius, 1781)) were collected in large numbers (more than 260 individuals each) during the study, whereas the most abundant curculionid (*Acalyptus carpini*) only attained a total of 87 individuals. The difference in abundance would imply that curculionid species on willow are either generally rarer, may have more rapid temporal turnover, or are less prone to outbreaks than chrysomelids. The alternative, and we believe less likely, hypothesis is that curculionids are intrinsically harder to catch in the sweep net than chrysomelids; we do note, however, that a reviewer of this paper believes weevils may be harder to capture in sweep nets as they sit further inside the shrub on woody branches. On the other hand, curculionids were more diverse with 74 species recorded in our samples versus only 34 species of chrysomelid (Canty et al. 2016, Canty et al. 2019). As curculionids are well known as a hyperdiverse group (Oberprieler et al. 2007) the higher diversity is hardly surprising.

## Usage rights

### Use license

Creative Commons Public Domain Waiver (CC-Zero)

## Data resources

### Data package title

*Salix* transect of Europe: records of willow-associated weevils

### Number of data sets

1

### Data set 1.

#### Data set name

*Salix* transect of Europe: records of willow-associated weevils

#### Number of columns

20

#### 

**Data set 1.** 

Column label	Column description
occurrenceID	An identifier for the Occurrence (as opposed to a particular digital record of the occurrence).
basisOfRecord	The specific nature of the data record.
recordedBy	A list (concatenated and separated) of names of people, groups or organisations responsible for recording the original Occurrence.
individualCount	The number of individuals represented present at the time of the Occurrence.
lifeStage	The age class or life stage of the biological individual(s) at the time the Occurrence was recorded.
samplingProtocol	The name of, reference to, or description of the method or protocol used during an Event.
eventDate	The date-time or interval during which an Event occurred.
locationID	An identifier for the set of location information (data associated with dcterms:Location).
country	The name of the country or major administrative unit in which the Location occurs.
minimumElevationInMeters	The lower limit of the range of elevation (altitude, usually above sea level), in metres.
maximumElevationInMeters	The upper limit of the range of elevation (altitude, usually above sea level), in metres.
decimalLatitude	The geographic latitude (in decimal degrees, using the spatial reference system given in geodeticDatum) of the geographic centre of a Location.
decimalLongitude	The geographic longitude (in decimal degrees, using the spatial reference system given in geodeticDatum) of the geographic centre of a Location.
geodeticDatum	The ellipsoid, geodetic datum or spatial reference system (SRS) upon which the geographic coordinates given in decimalLatitude and decimalLongitude are based.
identifiedBy	A list (concatenated and separated) of names of people, groups or organisations who assigned the Taxon to the subject.
dateIdentified	The date on which the subject was identified as representing the Taxon.
scientificName	The full scientific name, with authorship and date information, if known.
identificationQualifier	A brief phrase or a standard term ("cf.", "aff.") to express the determiner's doubts about the Identification.
verbatimTaxonRank	The taxonomic rank of the most specific name in the scientificName as it appears in the original record.
taxonRank	The taxonomic rank of the most specific name in the scientificName.

## Supplementary Material

9E494813-5705-5C36-BE14-4FBDA2524F21 10.3897/BDJ.8.e52881.suppl1 Supplementary material 1 Salix transect of Europe records of willow-associated weevils Data type Data set File: oo_397410.txt https://binary.pensoft.net/file/397410 Roy Canty, Enrico Ruzzier, Quentin C. Cronk, Diana M. Percy

## Figures and Tables

**Table 1.** Basic site details. See Cronk et al. (2015) for further details and Suppl. material 1.

**SITE**#	**Country**	**Lat N**	**Long E**	**Alt (m)**	**Date of collection**
1	Greece	38.80007	22.4629	37	21-iv-2015
2	Greece	38.902	22.31015	33	21-iv-2015
3	Greece	39.306694	22.528323	177	22-iv-2015
4	Greece	40.032685	22.175437	534	22-iv-2015
5	Greece	41.113317	23.273893	31	23-iv-2015
6	Bulgaria	41.412468	23.318609	90	23-iv-2015
7	Bulgaria	42.165622	22.998141	392	24-iv-2015
8	Bulgaria	42.923989	23.810563	339	24-iv-2015
9	Bulgaria	43.739343	23.966755	35	24-iv-2015
10	Romania	44.260343	23.786781	81	25-iv-2015
11	Romania	44.961981	23.190337	172	25-iv-2015
12	Romania	45.510676	22.737225	556	26-iv-2015
13	Romania	46.518504	21.512839	102	26-iv-2015
14	Hungary	46.700744	21.31268	94	27-iv-2015
15	Hungary	47.665648	21.261768	91	27-iv-2015
16	Hungary	48.374291	20.725264	148	28-iv-2015
17	Poland	49.463447	21.697255	385	28-iv-2015
18	Poland	50.470234	22.238372	157	29-iv-2015
19	Poland	50.673994	21.823391	141	29-iv-2015
20	Poland	51.775039	21.1971	101	30-iv-2015
20a	Poland	51.775039	21.1971	101	11-vi-2015
21	Poland	52.69398	21.8529	96	12-vi-2015
22	Poland	53.55483	22.30299	128	12-vi-2015
23	Poland	54.06943	23.11745	137	13-vi-2015
24	Lithuania	54.92583	23.7742	28	13-vi-2015
25	Lithuania	55.79557	24.56678	62	13-vi-2015
26	Latvia	56.71141	24.25162	23	14-vi-2015
27	Latvia	57.74963	24.4023	7	14-vi-2015
28	Estonia	58.42257	24.44063	18	15-vi-2015
29	Estonia	59.40289	24.93577	48	15-vi-2015
30	Finland	60.27299	24.65843	33	16-vi-2015
31	Finland	61.09965	25.6282	84	16-vi-2015
32	Finland	62.04962	26.12369	174	17-vi-2015
33	Finland	63.01589	25.80457	139	17-vi-2015
34	Finland	64.05074	25.52664	91	17-vi-2015
35	Finland	64.61287	25.53805	58	18-vi-2015
36	Finland	65.32835	25.29175	1	18-vi-2015
37	Finland	66.24947	23.8945	51	19-vi-2015
38	Finland	67.21253	24.12629	160	19-vi-2015
39	Finland	67.91183	23.63411	233	19-vi-2015
40	Norway	68.8138	23.26658	374	20-vi-2015
41	Norway	69.72487	23.40581	289	20-vi-2015
42	Norway	70.65234	23.66583	67	21-vi-2015

**Table 2.** Sequences generated during this study with site number along the transect, and GenBank accession numbers provided for cytochrome oxidase 1 (COI) and cytochrome B (cytB) gene regions included in analyses (Figs 3, 4). See Table 4 for taxonomic authorities.

**Species**	**Site**	**COI**	**cytB**
*Acalyptus carpini*	7	MN607603	MN607646
*Acalyptus carpini*	14	MN607604	MN607647
*Acalyptus carpini*	20	MN607605	MN607648
*Acalyptus carpini*	27	MN607606	MN607649
*Acalyptus carpini*	32	MN607607	MN607650
*Acalyptus carpini*	38	MN607608	MN607651
*Isochnus flagellum*	39	MN607613	MN607656
*Isochnus foliorum*	28	MN607615	MN607658
*Isochnus foliorum*	29	MN607609	MN607652
*Isochnus foliorum*	36	MN607610	MN607653
*Isochnus foliorum*	37	MN607611	MN607654
*Isochnus foliorum*	38	MN607612	MN607655
*Isochnus foliorum*	42	MN607614	MN607657
*Isochnus sequensi*	8	-	MN607663
*Isochnus sequensi*	14	-	MN607662
*Isochnus sequensi*	20	-	MN607661
*Isochnus sequensi*	21	-	MN607660
*Isochnus sequensi*	22	MN607616	MN607659
*Melanapion minimum*	7	MN607622	MN607669
*Melanapion minimum*	11	MN607621	MN607668
*Melanapion minimum*	20	MN607620	MN607667
*Melanapion minimum*	21	MN607619	MN607666
*Melanapion minimum*	26	MN607618	MN607665
*Melanapion minimum*	28	MN607617	MN607664
*Phyllobius arborator*	22	MN607624	MN607671
*Phyllobius maculicornis*	24	MN607625	MN607672
*Phyllobius maculicornis*	26	MN607626	MN607673
*Phyllobius maculicornis*	29	MN607627	MN607674
*Phyllobius maculicornis*	35	MN607628	MN607675
*Phyllobius oblongus*	1	MN607629	MN607676
*Phyllobius oblongus*	4	MN607630	MN607677
*Phyllobius oblongus*	8	MN607631	MN607678
*Phyllobius oblongus*	12	MN607632	MN607679
*Phyllobius oblongus*	16	MN607633	MN607680
*Phyllobius oblongus*	31	MN607634	MN607681
*Phyllobius thalassinus*	15	MN607623	MN607670
*Rhamphus pulicarius*	20	-	MN607686
*Rhamphus pulicarius*	21	MN607639	MN607685
*Rhamphus pulicarius*	23	MN607638	MN607684
*Rhamphus pulicarius*	24	MN607637	MN607683
*Rhamphus pulicarius*	27	MN607636	-
*Rhamphus pulicarius*	28	MN607635	MN607682
*Tachyerges pseudostigma*	8	MN607644	MN607691
*Tachyerges pseudostigma*	16	MN607645	MN607692
*Tachyerges pseudostigma*	29	MN607641	MN607688
*Tachyerges pseudostigma*	37	MN607642	MN607689
*Tachyerges stigma*	2	MN607643	MN607690
*Tachyerges stigma*	23	MN607640	MN607687

**Table 3.** Previously published sequences obtained from GenBank and included in the analysis in Fig. 4. Taxonomic authorities are given for five taxa only sampled from GenBank. See Table 4 for taxonomic authorities for taxa sampled in this study.

**Species**	**GenBank**
*Acalyptus carpini*	KJ963255, KM448779, KJ202744, KJ202760, KJ203684, KJ203788
*Isochnus flagellum*	KU875304
*Isochnus foliorum*	KJ964448
*Isochnus sequensi*	KM443507, KM440769, KU914939, KR489841, KM449616, MG061165
*Melanapion minimum*	KJ967202, KY084065, KU910174
*Phyllobius arborator*	KM444121, KU917359, KM442278, KU918158, KU914021, KM450213
*Phyllobius betulinus* (Bechstein & Scharfenberg, 1805)	KU918630, KU914490, KU907012
*Phyllobius calcaratus* (Fabricius, 1792)	KU918134, KM449838, KU910170, KM442586, KU906623, KM443590, KM439992
*Phyllobius maculicornis*	KJ962100, KM451423, KU918601, KM444203, KM440389, KJ961942
*Phyllobius oblongus*	MF634782, MF635360, MF634673, MF633476, KC784036
*Phyllobius pomaceus* Gyllenhal, 1834	KU917534, KU912973, KM441444, KM446832, KJ963568, KJ963097, KJ962197, KM440340
*Phyllobius roboretanus* Gredler, 1882	KU907507
*Phyllobius virideaeris* (Laicharting, 1781)	KU910818, KU909724, KU906909, KU914286
*Rhamphus pulicarius*	KJ962692, KU914674, KU909870, KU917811, KM443697
*Tachyerges stigma*	KU908471, KJ961997, KJ962461, KU917995, KU918982, KM448429

**Table 4.** Species recorded, in order of number of sites. The first seven species form the most widespread and abundant group (see Table 5 for more details). Those weevils found at eight sites or more are classified into wide, central, northern and southern occurrence tendencies. Individual sites of occurrence are given for all species (with numbers of individuals in brackets if more than one); counts marked > indicate that not all individuals were counted.

**SPECIES [FAMILY**]	**Number of sites (S)**	**Number of individuals (N)**	**Abundance index (NxS)**	**Sites (with no. of individuals in brackets)**
*Acalyptus carpini* Fabricius, 1792 [Curculionidae]	15	87	**1305**	7(7), 8(4), 11(9), 12(4), 14(15), 15, 16(2), 17(27), 19, 20(6), 27(2), 28(2), 32, 37(4), 38(2) [wide]
*Tachyerges stigma* Germar, 1821 [Curculionidae]	13	26	**338**	2, 5, 6(3), 12, 23, 27, 30(2), 32(2), 33(8), 34, 35, 37(3), 38 [wide]
*Phyllobius oblongus* (Linnaeus, 1758) [Curculionidae]	11	31	**341**	1(8), 2(7), 3, 4, 8, 10(3), 12, 14(3), 15(4), 16, 31 [1-16 southern]
*Phyllobius maculicornis* Germar, 1824 [Curculionidae]	10	36	**360**	11(2), 15, 21, 24(2), 26(4), 27(17), 28(6), 29, 35, 36 [wide]
*Melanapion minimum* (Herbst, 1797) [Brentidae]	9	22	**198**	7, 11(2), 16(2), 17(4), 18(4), 20(2), 21(2), 26, 28(4) [central]
Phyllobius cf. pyri (Linnaeus, 1758) [*Curculionidae*]	9	21	**189**	11(5), 12(6), 15(2), 16, 17(2), 19(2), 28, 30, 36 [wide]
*Archarius salicivorus* (Paykull, 1792) [Curculionidae]	9	13	**117**	4, 7(2), 11(3), 14, 15, 16, 17, 25(2), 27 [south-central]
*Isochnus foliorum* (Müller, 1764) [Curculionidae]	8	40	**320**	28, 29, 30, 36(2), 37(3), 38(2), 41(5), 42(25) [northern]
*Rhamphus pulicarius* (Herbst, 1795) [Curculionidae]	8	29	**232**	20, 20a(13), 21(3), 22, 23, 24, 27, 28(8) [northern]
*Archarius crux* (Fabricius, 1776) [Curculionidae]	8	14	**112**	11, 12(2), 13(2), 17(2), 20, 20a(2), 21(2), 27(2) [central]
*Tachyerges pseudostigma* (Tempère, 1982) [Curculionidae]	8	11	**88**	8, 11(2), 16, 18(2), 25, 26, 29, 37(2) [north-central]
*Temnocerus tomentosus* (Gyllenhal, 1839) [Attelabidae]	7	11	**77**	6, 20, 20a(2), 23(2), 28(2), 33(2), 36
*Tachyerges salicis* (Linnaeus, 1758) [Curculionidae]	7	9	**63**	11, 16, 28, 29, 32(2), 37(2), 39
*Polydrusus flavipes* (De Geer, 1775) [Curculionidae]	6	80	**480**	13, 20, 20a(2), 21(73), 28, 31(2)
*Isochnus sequensi* (Stierlin, 1894) [Curculionidae]	6	40	**240**	8(21), 14, 20, 20a(10), 21(4), 22(3)
*Ellescus bipunctatus* (Linnaeus, 1758) [Curculionidae]	5	6	**30**	7, 12, 33, 37(2), 40
*Dorytomus taeniatus* (Fabricius, 1781) [Curculionidae]	4	14	**56**	12(6), 18(2), 20a(3), 38(3)
*Phyllobius glaucus* (Scopoli, 1763) [Curculionidae]	4	6	**24**	8(3), 13, 20, 27
*Tachyerges decoratus* (Germar, 1821) [Curculionidae]	4	5	**20**	12, 17(2), 30, 37
*Polydrusus prasinus* (Olivier, 1790) [Curculionidae]	3	9	**27**	1(7), 2, 3
Isochnus cf. angustifrons (West, 1916) [Curculionidae]	3	5	**15**	19, 27, 39(3)
*Phyllobius viridicollis* (Fabricius, 1801) [Curculionidae]	3	3	**9**	3, 26, 27
Protapion cf. fulvipes (Geoffroy in Fourcroy, 1785) [Brentidae]	3	4	**12**	8, 11(2), 27
Dorytomus cf. salicinus (Gyllenhal, 1827) [Curculionidae]	2	12	**24**	17, 39(11)
Ellescus cf. scanius (Paykull, 1792) [Curculionidae]	2	10	**20**	17(9), 20
*Polydrusus picus* (Fabricius, 1792) [Curculionidae]	2	7	**14**	20, 20a(6)
Dorytomus cf. dejeani Faust, 1882 [Curculionidae]	2	4	**8**	17, 20a(3)
*Oxystoma* sp. [Brentidae]	2	4	**8**	23(3), 37
Phyllobius cf. pomaceus (Gyllenhal, 1834) [Curculionidae]	2	3	**6**	27, 35(2)
*Protapion schoenherri* (Boheman, 1839) [Brentidae]	2	3	**6**	7, 11(2)
*Phyllobius argentatus* (Linnaeus, 1758) [Curculionidae]	2	2	**4**	30, 32
*Protapion* sp. [Brentidae]	2	2	**4**	13, 17
*Byctiscus betulae* (Linnaeus, 1758) [Attelabidae]	2	2	**4**	6, 24
Polydrusus cf. pilosus (Gredler, 1866) [Curculionidae]	2	2	**4**	21, 36
*Polydrusus impar* Des Gozis, 1882 [Curculionidae]	2	2	**4**	17, 20a
*Phyllobius arborator* (Herbst, 1797) [Curculionidae]	2	2	**4**	21, 22
*Dorytomas rufatus* (Bedel, 1888) [Curculionidae]	2	2	**4**	15, 21
Scolytinae sp. [Curculionidae]	2	2	**4**	11, 33
Polydrusus cf. pterygomalis Boheman, 1840 [Curculionidae]	1	20	**20**	10(>20)
*Isochnus flagellum* (Ericson, 1902) [Curculionidae]	1	7	**7**	39(7)
*Chlorophanus viridis* (Linnaeus, 1758) [Curculionidae]	1	5	**5**	21(5)
*Phyllobius viridiaeris* (Laicharting, 1781) [Curculionidae]	1	3	**3**	20a(3)
*Isochnus populicola* (Silfverberg, 1977) [Curculionidae]	1	1	**1**	11
Dorytomus cf. melanophthalmus (Paykull, 1792) [Curculionidae]	1	1	**1**	21
*Ellescus infirmus* (Herbst, 1792) [Curculionidae]	1	1	**1**	37
*Tanymecus* sp. [Curculionidae]	1	1	**1**	15
Anthonomus cf. conspersus Desbrochers, 1868 [Curculionidae]	1	1	**1**	16
*Betulapion* sp. [Brentidae]	1	1	**1**	11
Ceutorhynchus cf. assimilis (Paykull, 1792) [Curculionidae]	1	1	**1**	8
Coeliodes cf. rubicundus (Herbst, 1795) [Curculionidae]	1	1	**1**	39
Deporaus cf. mannerheimi (Hummel, 1823) [Attelabidae]	1	1	**1**	12
Dorytomus cf. affinis (Paykull, 1800) [Curculionidae]	1	1	**1**	41
Dorytomus cf. salicis Walton, 1851 [Curculionidae]	1	1	**1**	20
Dorytomus cf. tortrix (Linnaeus, 1761) [Curculionidae]	1	1	**1**	20a
Dorytomus cf. tremulae (Fabricius, 1787) [Curculionidae]	1	1	**1**	6
Eutrichapion cf. punctigerum (Paykull, 1792) [Brentidae]	1	1	**1**	30
*Hylobius abietis* (Linnaeus, 1758) [Curculionidae]	1	1	**1**	36
*Lepyrus palustris* (Scopoli, 1763) [Curculionidae]	1	1	**1**	12
Nanophyes cf. marmoratus (Goeze,1777) [Brentidae]	1	1	**1**	15
*Perapion* sp. [Brentidae]	1	1	**1**	42
*Polydrusus ruficornis* (Bonsdorff, 1785) [Curculionidae]	1	1	**1**	35
*Orchestes testaceus* (Müller, O.F., 1776) [Curculionidae]	1	1	**1**	32
Sitona cf. lineatus (Linnaeus, 1758) [Curculionidae]	1	1	**1**	34
*Stenopterapion* sp. [Brentidae]	1	1	**1**	11
*Neliocarus nebulosus* (Stephens, 1831) [Curculionidae]	1	1	**1**	36
Neocoenorrhinus cf. aeneovirens (Marsham, 1802) [Attelabidae]	1	1	**1**	16
*Magdalis phlegmatica* (Herbst, 1797) [Curculionidae]	1	1	**1**	36
*Phyllobius thalassinus* Gyllenhal, 1834 [Curculionidae]	1	1	**1**	15
*Protapion varipes* (Germar, 1817) [Brentidae]	1	1	**1**	7
*Anthribus nebulosus* Forster, 1770 [Anthribidae]	1	1	**1**	20
*Dissoleucas niveirostris* (Fabricius, 1798) [Anthribidae]	1	1	**1**	8
Protapion cf. ruficroides (Dieckmann, 1973) [Brentidae]	1	1	**1**	28

**Table 5.** Abundance of widespread (>8 sites) species at particular sites. Counts of individuals are given for all samples. Abbreviations: Tot. (wide) = Total individuals at sites (widespread species); Tot. (all) = Total individuals at sites (all species); N. spp. = number of weevil species at sites.

**Site**	**Acal. carp.**	**Tach. stig.**	**Phyl. obl.**	**Phyl. mac.**	**Mel. min.**	**Phyl. pyr.**	**Arch. salic.**	**Tot. (wide)**	**Tot. (all)**	**N. spp.**
**1**			8					8	15	2
**2**		1	7					8	9	3
**3**			1					1	3	3
**4**			1				1	2	2	2
**5**		1						1	1	1
**6**		3						3	6	4
**7**	7				1		2	10	13	6
**8**	4		1					5	33	8
**9**								0	0	0
**10**			3					3	23	2
**11**	9			2	2	5	3	21	33	14
**12**	4	1	1			6		12	24	10
**13**								0	6	5
**14**	15		3				1	19	20	4
**15**	1		4	1		2	1	9	13	9
**16**	2		1		2	1	1	7	11	9
**17**	27				4	2	1	34	52	12
**18**					4			4	8	3
**19**	1					2		3	4	3
**20**	6				2			8	18	12
**20a**								0	45	11
**21**				1	2			3	94	11
**22**								0	5	3
**23**		1						1	7	4
**24**				2				2	4	3
**25**							2	2	3	2
**26**				4	1			5	7	4
**27**	2	1		17			1	21	29	11
**28**	2			6	4	1		13	27	10
**29**				1				1	4	4
**30**		2				1		3	7	5
**31**			1					1	3	2
**32**	1	2						3	7	5
**33**		8						8	12	4
**34**		1						1	2	2
**35**		1		1				2	6	5
**36**				1		1		2	8	7
**37**	4	3						7	19	9
**38**	2	1						3	8	4
**39**								0	23	5
**40**								0	1	1
**41**								0	6	2
**42**								0	26	2
TOT	87	26	31	36	22	21	13	236	647	

**Table 6.** Measurements of representative individuals of some common species to show variation.

**Species**	**Sites**	**Elytra colour on scored individuals**	**Elytra length (mm)**	**Elytra width at shoulder (mm)**	**Pronotal length (mm)**	**Pronotal width at base (mm)**
*Acalyptus carpini*	7,14,20, 27,32,38	165B,165C,203C	1.6-1.7	1.0-1.1	0.6	0.8
*Isochnus foliorum*	29,36,37,38,42	203B	0.9-1.3	0.5-0.7	0.3-0.4	0.4
*Isochnus sequensi*	8,14,20, 21,22	203B	1.3-1.7	0.7-0.9	0.4	0.4-0.5
*Melanapion minimum*	7,11,20, 21,26,28	203B	1.1-1.4	0.6-0.7	0.4-0.5	0.4-0.5
*Phyllobius maculicornis*	24,26,29,35	Elytra:203A; Scales:101C,121C,104D,115D	3.4-3.9	1.7-1.9	0.9-1.2	1.1-1.2
*Phyllobius oblongus*	1,4,8,12,16,31	164A,163B,165B,164C,162D,203D	3.2-3.5	1.4-1.6	0.9	0.9
*Rhamphus pulicarius*	20,21,23,24,27,28	203B	1.1-1.4	0.5-0.7	0.4	0.4-0.6
*Tachyerges pseudostigma*	8,16,29, 37	203C	1.7-2.1	0.9-1.2	0.5-0.7	0.6-0.8
